# Bidirectional 2-sample Mendelian randomization study of the causal relationship between hypothyroidism and cardiovascular diseases

**DOI:** 10.1097/MD.0000000000045889

**Published:** 2025-11-21

**Authors:** Jianing Chi, Sheng Qiu, Wenqiang Ma, Guanyu Yan, Yanhua Xue

**Affiliations:** a Naval Submarine Academy, Qingdao, China.

**Keywords:** coronary heart disease, heart failure, hypertension, hypothyroidism, Mendelian randomization, myocardial infarction

## Abstract

A bidirectional 2-sample Mendelian randomization (MR) analysis was used to investigate the causal relationship between hypothyroidism and multiple types of cardiovascular diseases (CVDs). Single nucleotide polymorphisms strongly associated with hypothyroidism and 4 CVDs (myocardial infarction, coronary artery disease, heart failure [HF], hypertension) were extracted from genome-wide association study summary data as instrumental variables, excluding linkage disequilibrium. First, a forward MR analysis assessed causal effects of hypothyroidism on cardiovascular outcomes using inverse-variance weighted, weighted median, MR-Egger regression, simple mode, and weighted mode methods. Heterogeneity was evaluated via inverse-variance weighted and MR-Egger *Q*-tests, pleiotropy via Egger intercept test, and sensitivity via leave-one-out analysis. Reverse MR tested causality from CVDs to hypothyroidism. A bidirectional MR framework ensured robustness, with comprehensive statistical validations (heterogeneity, pleiotropy, sensitivity) to minimize confounding bias and confirm reliability of causal inferences. Genetically predicted hypothyroidism causally increased risks of myocardial infarction (odds ratio [OR] = 3.003, *P* = .002), coronary heart disease (OR = 2.780, *P* = .001), HF (OR = 1.892, *P* = .009), and hypertension (OR = 1.102, *P* = .024). Reverse MR revealed HF reciprocally elevated hypothyroidism risk (OR = 1.006, *P* = .040). Analyses confirmed absence of pleiotropy (all *P* > .05) and robustness through leave-one-out sensitivity analysis. Causal relationships remained consistent across sensitivity methods, supporting thyroid dysfunction as cardiovascular risk modifier in bidirectional pathways. This study affirms that hypothyroidism heightens the susceptibility to multiple types of CVDs, and establishes a causal link between HF and hypothyroidism.

## 1. Introduction

With the extension of life expectancy and the acceleration of population aging, thyroid diseases have gradually emerged as one of the prevalent geriatric disorders. According to statistics, over 50% of elderly individuals in China suffer from thyroid diseases, with hypothyroidism being the most common.^[1]^ The prevalence of hypothyroidism increases progressively with age. Hypothyroidism is a systemic hypometabolic syndrome caused by reduced synthesis and secretion of thyroid hormones (TH) or diminished tissue effects, with over 90% of cases being primary hypothyroidism. In elderly patients, hypothyroidism often presents with an insidious onset, slow disease progression, and atypical clinical manifestations that are easily mistaken for signs of normal aging. Furthermore, geriatric hypothyroidism frequently coexists with multiple metabolic disorders, such as coronary heart disease (CHD), hypertension (HTN), and diabetes mellitus, complicating diagnosis and increasing the risk of missed or misdiagnosis. These factors significantly impair the health status and quality of life in the elderly population.^[2]^

Cardiovascular diseases (CVDs) are the leading cause of global mortality and disability, claiming approximately 17.9 million lives annually and imposing an escalating burden on health and society.^[3]^ As the cardiovascular system is one of the primary targets of TH, extensive research has focused on the potential association between hypothyroidism and CVDs. Most studies confirm that geriatric hypothyroidism contributes to dyslipidemia, accelerated atherosclerosis, endothelial dysfunction, and left ventricular dysfunction, thereby elevating the risk of CVDs incidence and mortality.^[4–7]^ However, Feng^[8]^ in a prospective analysis of 140 elderly individuals, suggested that abnormal thyroid-stimulating hormone (TSH) levels do not necessarily increase cardiovascular event risk. Meanwhile, Tsai^[9]^ conducted a systematic review and meta-analysis of 27 cohort studies involving 1.11 million patients, revealing that while hypothyroidism patients exhibited higher all-cause mortality compared to euthyroid individuals, no significant difference was observed in cardiovascular mortality. It is critical to note that these findings primarily derive from observational studies with case-control designs, which are limited by temporal sequence ambiguity, measurement errors, potential biases, and confounding factors. Consequently, these studies have not conclusively elucidated the true causal relationship between hypothyroidism and CVDs.^[10]^

Two-sample Mendelian randomization (TSMR) has been widely employed in recent years to investigate causal relationships in epidemiology from a genetic perspective. This method utilizes genetic variants as proxies for exposures, enabling the assessment of causal effects of these exposures on outcomes and advancing research into disease pathogenesis.^[11–13]^ Based on Mendel’s law of segregation, alleles segregate during gamete formation and combine randomly during fertilization.^[14]^ Leveraging this principle, the TSMR approach uses single nucleotide polymorphisms (SNPs) within genes as instrumental variables (IVs), effectively mitigating confounding biases inherent in traditional observational studies.^[15–18]^ Previous MR studies on thyroid function and CVDs were conducted in multiethnic populations and predominantly focused on ischemic heart disease as a single endpoint.^[19–21]^ To date, no bidirectional MR analysis has explored the relationship between hypothyroidism and a broader spectrum of CVDs, including HTN and heart failure (HF). Therefore, this study employs bidirectional TSMR analysis to investigate potential bidirectional causality between hypothyroidism and multiple CVDs, specifically myocardial infarction (MI), CHD, HF, and HTN, and to underscore the importance of monitoring cardiovascular function in elderly individuals with hypothyroidism.

## 2. Materials and methods

### 2.1. Study design

**Figure 1.**
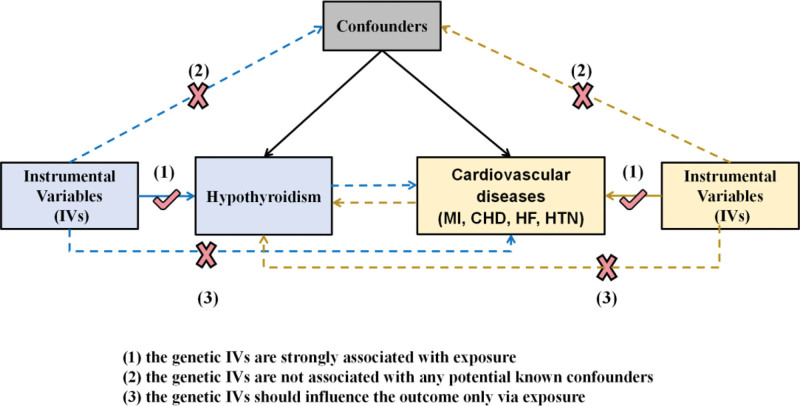
Model of the bidirectional 2-sample Mendelian randomization analysis. (1), (2) and (3) are the research hypotheses. CHD = coronary heart disease, HF = heart failure, HTN = hypertension, MI = myocardial infarction.

This study utilized a TSMR design to investigate the causal relationship between hypothyroidism and CVDs using IVs. The analysis was grounded in 3 core assumptions: a strong association exists between the genetic IVs and the exposure (hypothyroidism); the genetic IVs are not confounded by known or unknown factors; and the genetic IVs influence the outcome (CVDs) solely through their effect on the exposure, with no pleiotropic pathways. A conceptual framework of this MR study is illustrated in Figure 1. As the analysis relied on publicly available data, no additional ethical approval or informed consent was required.

### 2.2. Data sources

The genome-wide association study (GWAS) data for both exposure and outcomes in this study were obtained from the GWAS summary statistics repository (https://gwas.mrcieu.ac.uk/). Hypothyroidism-associated SNPs were derived from the study by Loh,^[22]^ which included 4,73,703 participants and identified over 11 million SNPs. For the analysis of 4 common CVDs: MI and CHD GWAS data were sourced from the CARDIoGRAMplusC4D consortium, comprising 43,676 cases and 1,28,199 controls for MI, and 60,801 cases and 1,23,504 controls for CHD; HF and HTN data were extracted from the EBI GWAS database, specifically studies by Shah^[23]^ (9,77,323 participants) and Dönertaş^[24]^ (4,84,598 participants), respectively. A summary of all datasets included in this study is provided in Table 1.

**Table 1:** GWAS data information on exposures and outcomes in 2-sample MR studies.

Exposure/outcome	ID	Sample size	SNPs	Race	Consortium of study
Hypothyroidism	ebi-a-GCST90029022	1,71,875	92,89,492	European	EBI
Myocardial infarction	ieu-a-798	27,541	1,63,80,466	European	CARDIoGRAMplusC4D
Coronary heart disease	ieu-a-7	6618	75,93,175	European	CARDIoGRAMplusC4D
Heart failure	ebi-a-GCST009541	4,82,730	1,78,91,936	European	EBI
Hypertension	ebi-a-GCST90038604	38,589	1,56,632	European	EBI

EBI = European Bioinformatics Institute, GWAS = genome-wide association study, ID = identity document, SNP = single nucleotide polymorphism.

### 2.3. Selection of instrumental variables

IVs were selected in accordance with the 3 core assumptions of MR studies. First, we identified SNPs strongly associated with hypothyroidism, MI, CHD, HF, and HTN at genome-wide significance (*P* < 5 × 10^8^) as IVs. Second, to ensure independence among SNPs and eliminate linkage disequilibrium (LD), clumping was performed using standard parameters (*R*^2^ < 0.001, clumping window = 10,000 kb). Third, SNPs associated with outcomes (pleiotropic effects) were excluded using the GWAS Catalog, and those with minor allele frequencies <0.01 were removed. To harmonize exposure and outcome datasets, palindromic SNPs and ambiguous allele-mismatched variants were excluded.^[25]^ Additionally, prior to MR analysis, we calculated the *F* statistic to evaluate IV strength. An *F*-statistic > 10 was required, indicating robust instrument strength and minimizing weak instrument bias in MR estimates.^[26,27]^

### 2.4. Statistical analysis

We employed multiple analytical approaches, including inverse-variance weighted (IVW), MR-Egger regression, weighted median (WM), simple mode, and weighted mode methods, to estimate the causal relationship between hypothyroidism and susceptibility to CVDs. The IVW method served as the primary MR analysis, which aggregates weighted averages of Wald ratio estimates for the causal effect of each genetic variant. This method quantifies the influence of each SNP on standardized log-transformed exposure levels and provides robust causal effect estimates.^[14]^

Following MR analysis, a series of sensitivity analyses were conducted to evaluate validity and robustness, including heterogeneity testing, pleiotropy assessment, and leave-one-out sensitivity analysis. Heterogeneity among IVs was assessed using Cochran’s *Q* statistic, with *P* < .05 indicating significant heterogeneity. In such cases, causal effects were estimated via IVW random-effects models. Pleiotropy was evaluated by testing the deviation of the MR-Egger regression intercept from zero.^[28]^ Additionally, leave-one-out analysis was performed by iteratively excluding individual SNPs to assess their influence on the pooled effect estimates.^[29]^ Forest plots were generated to visualize effect estimates between genetic variants and hypothyroidism or CVDs, with combined effects calculated using MR-Egger regression and IVW.

All statistical analyses were performed using the “TwoSampleMR” package in R software (version 4.2.2). A 2-sided *P* < .05 was considered statistically significant.

## 3. Results

### 3.1. Causal effect of hypothyroidism on CVDs

#### 3.1.1. IVs selection

Using hypothyroidism as the exposure and phenotypes of CVDs as outcomes, we identified 129 SNPs significantly associated with hypothyroidism following correlation screening and LD pruning. Subsequently, SNPs associated with outcomes and palindromic SNPs with ambiguous allele orientation were excluded. Ultimately, 106 SNPs were screened as an IV for the causal association between hypothyroidism and MI; 106 SNPs were used as IVs for the causal association between hypothyroidism and CHD; 107 SNPs were used as IVs for the causal association between hypothyroidism and HF; and 109 SNPs were used as IVs for the causal association between hypothyroidism and HTN for MR analysis. All retained SNPs exhibited an *F*-statistic > 10, confirming their adequacy as strong genetic instruments and minimizing weak IV bias in causal effect estimation.

#### 3.1.2. MR analysis results

**Figure 2.**
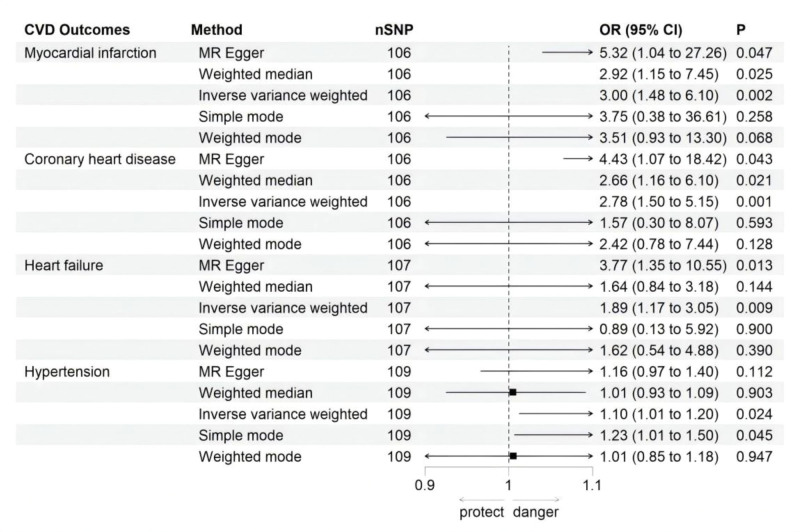
Forest plot for MR analysis causal effect of hypothyroidism on CVDs phenotype. CI = confidence interval, CVDs = cardiovascular diseases, MR = Mendelian randomization, OR = odds ratio, SNP = single nucleotide polymorphism.

**Figure 3.**
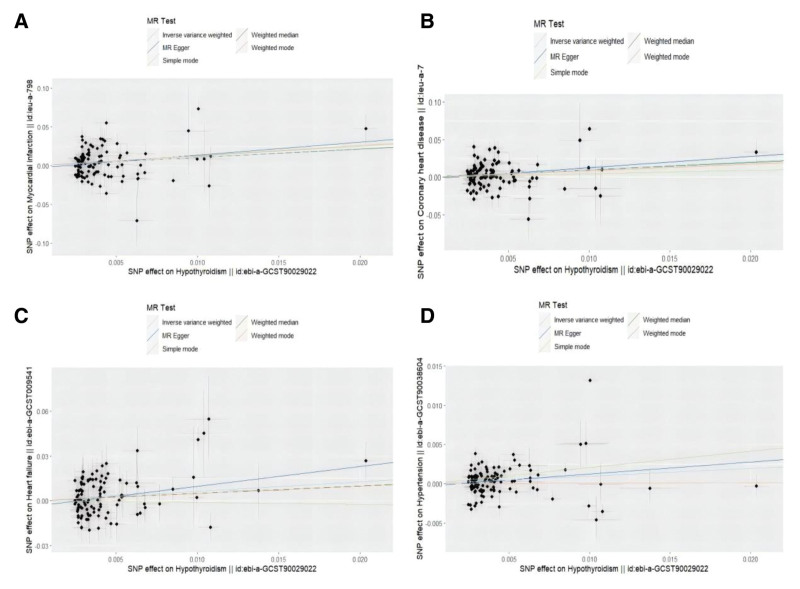
Scatter plots for MR analyses of the causal effect of hypothyroidism on CVDs phenotype. (A) hypothyroidism-MI; (B) hypothyroidism-CHD; (C) hypothyroidism-HF; and (D) hypothyroidism-HTN. Each point represents an individual SNP. The slope of the regression line indicates the causal effect estimate of exposure (hypothyroidism) on outcome (CVDs phenotypes) from MR methods. *X*-axis: genetic association with hypothyroidism (OR). *Y*-axis: genetic association with CVDs phenotypes (OR). CHD = coronary heart disease, CVDs = cardiovascular diseases, HF = heart failure, HTN = hypertension, MR = Mendelian randomization, MI = myocardial infarction, OR = odds ratio, SNP = single nucleotide polymorphism.

MR analyses demonstrated a causal association between hypothyroidism and MI, CHD, HF, and HTN, as illustrated in Figure 2. Figure 3 depicts the effect estimates of SNPs on exposure (hypothyroidism) and outcomes (CVDs). The random-effects IVW method revealed that genetically predicted hypothyroidism was significantly associated with an elevated risk of MI (odds ratio [OR] = 3.004, 95% confidence interval [CI]: 1.478–6.105, *P* = .002). Consistent risk estimates were observed in MR-Egger (OR = 5.325, 95% CI: 1.040–27.258, *P* = .047) and WM analyses (OR = 2.921, 95% CI: 1.146–7.446, *P* = .025). Similarly, hypothyroidism showed a causal relationship with increased CHD risk (IVW: OR = 2.780, 95% CI: 1.502–5.146, *P* = .001), with concordant results from MR-Egger and WM methods. For HF, both IVW (OR = 1.892, 95% CI: 1.172–3.054, *P* = .009) and MR-Egger (OR = 3.767, 95% CI: 1.345–10.545, *P* = .013) identified hypothyroidism as a risk factor. Additionally, the analysis suggested a potential causal link between hypothyroidism and HTN (IVW: OR = 1.102, 95% CI: 1.013–1.199, *P* = .024).

### 3.2. Sensitivity analysis

**Figure 4.**
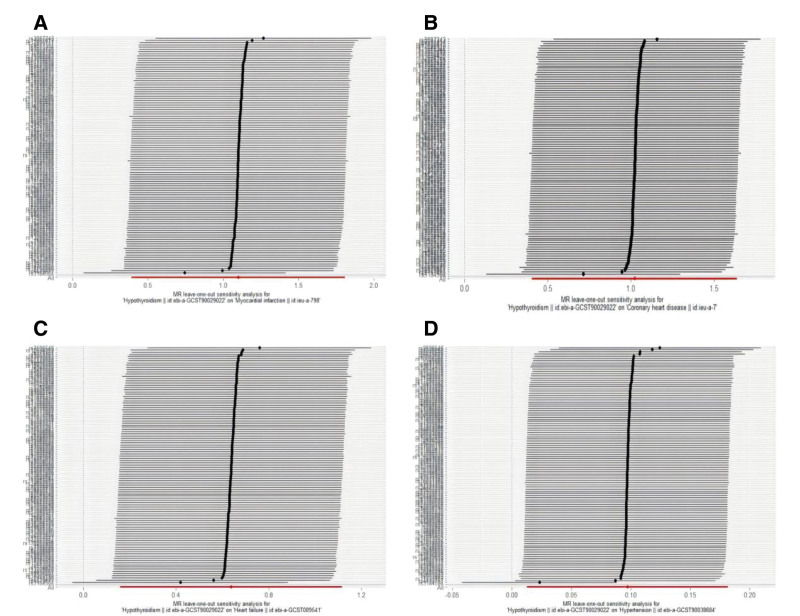
Leave-one-out sensitivity analysis between hypothyroidism and CVDs phenotype. (A) hypothyroidism-MI; (B) hypothyroidism-CHD; (C) hypothyroidism-HF; and (D) hypothyroidism-HTN. Each line represents the causal estimate when sequentially excluding 1 SNP at a time. *X*-axis: estimated causal effect of hypothyroidism on CVDs phenotypes risk. *Y*-axis: SNP identifier. The horizontal dashed line indicates the primary MR estimate. CHD = coronary heart disease, CVDs = cardiovascular diseases, HF = heart failure, HTN = hypertension, MR = Mendelian randomization, MI = myocardial infarction, SNP = single nucleotide polymorphism.

Heterogeneity among IVs was assessed using the IVW method and MR-Egger regression. Cochran’s *Q* test (Table 2) indicated significant heterogeneity between hypothyroidism and MI, CHD, HF, and HTN (*P* < .05). To mitigate the impact of heterogeneity, random-effects models were applied in the analyses. Horizontal pleiotropy was evaluated via the MR-Egger intercept test (Table 2), which revealed no significant pleiotropic effects for hypothyroidism with MI (*P* = .447), CHD (*P* = .479), HF (*P* = .142), or HTN (*P* = .517), suggesting that IVs influenced outcomes primarily through the exposure pathway rather than alternative mechanisms. Furthermore, leave-one-out sensitivity analysis (Fig. 4) demonstrated that sequentially excluding individual SNPs had minimal impact on the pooled effect estimates for hypothyroidism in relation to MI, CHD, HF, and HTN, confirming the robustness of the MR findings.

**Table 2:** Sensitivity test of the mendelian randomization analysis between hypothyroidism and CVDs phenotypes.

Exposure	Outcome	Heterogeneity test			Horizontal pleiotropy	
		Methods	*Q*	*P*	Egger_intercept	*P*
Hypothyroidism	Myocardial infarction	IVW	188.713	1.01E−06	−0.003	.447
		MR-Egger	187.662	9.52E−07		
Hypothyroidism	Coronary heart disease	IVW	173.537	2.95E−05	−0.002	.479
		MR-Egger	172.697	2.69E−05		
Hypothyroidism	Heart failure	IVW	166.278	1.67E−04	−0.004	.142
		MR-Egger	162.887	2.54E−04		
Hypothyroidism	Hypertension	IVW	493.124	1.46E−50	−0.0003	.517
		MR-Egger	491.185	1.46E−50		
Myocardial infarction	Hypothyroidism	IVW	514.824	5.90E−95	0.001	.285
		MR-Egger	486.948	7.85E−90		
Coronary heart disease	Hypothyroidism	IVW	487.364	6.26E−80	0.001	.105
		MR-Egger	452.686	1.64E−73		
Heart failure	Hypothyroidism	IVW	7.442	.490	0.0005	.396
		MR-Egger	6.625	.469		
Hypertension	Hypothyroidism	IVW	449.540	2.34E−18	0.0002	.189
		MR-Egger	445.947	4.16E−18		

*P* value < .05 is considered statistically significant.

CVD = cardiovascular disease; IVW = inverse-variance weighted.

### 3.3. Reverse MR analysis

#### 3.3.1. IVs selection

In the reverse MR analysis, where phenotypes of CVDs served as exposures and hypothyroidism as the outcome, we identified 26, 41, 12, and 277 SNPs associated with MI, CHD, HF, and HTN, respectively, following exposure-correlation screening and LD pruning. After excluding outcome-associated SNPs and palindromic SNPs with ambiguous allele orientation, 23, 38, 9, and 218 SNPs were retained as IVs for reverse MR analysis. All SNPs exhibited an *F*-statistic > 10, reinforcing the validity and reliability of our analysis.

#### 3.3.2. MR analysis results

**Figure 5.**
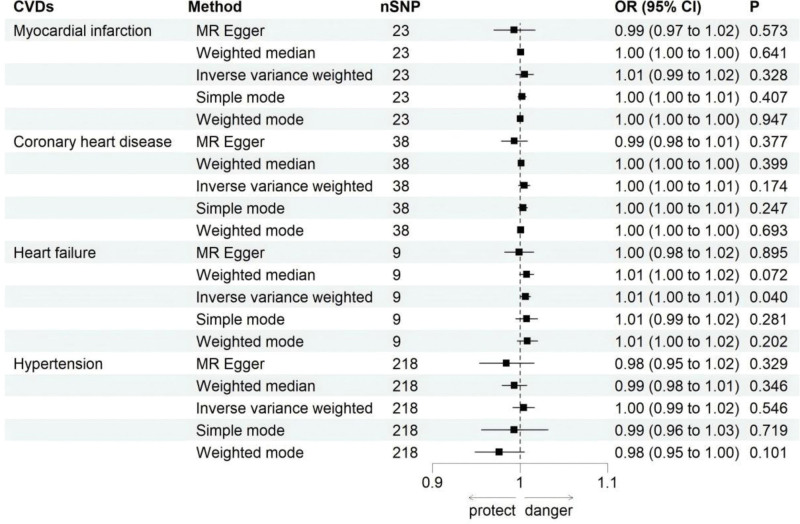
Forest plot for MR analysis causal effect of CVDs phenotype on hypothyroidism. CI = confidence interval, CVDs = cardiovascular diseases, MR = Mendelian randomization, OR = odds ratio, SNP = single nucleotide polymorphism.

**Figure 6.**
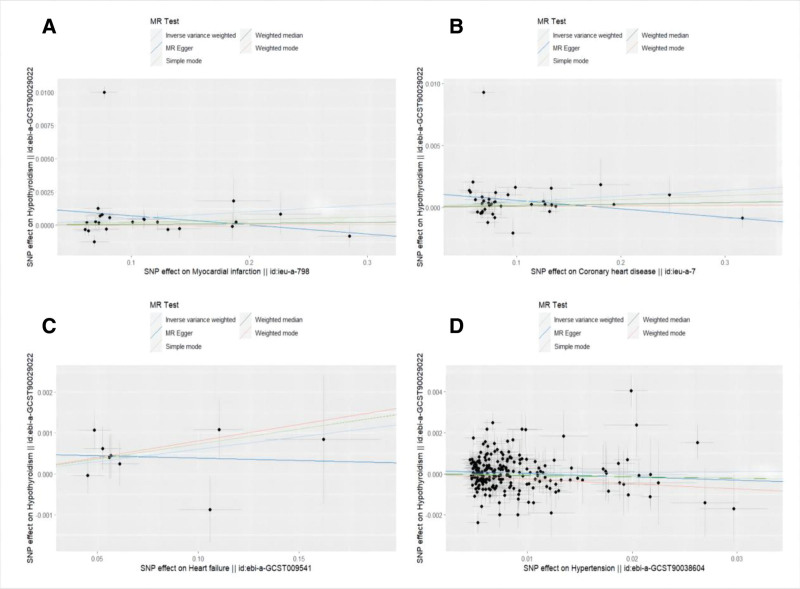
Scatter plots for MR analyses of the causal effect of CVDs phenotype on hypothyroidism. (A) MI-hypothyroidism; (B) CHD-hypothyroidism; (C) HF-hypothyroidism; and (D) hypertension-hypothyroidism. Each point represents an individual SNP. The slope of the regression line indicates the causal effect estimate of exposure (CVDs phenotypes) on outcome (hypothyroidism) from MR methods. *X*-axis: genetic association with CVDs phenotypes (OR). *Y*-axis: genetic association with hypothyroidism (OR). CHD = coronary heart disease, CVDs = cardiovascular diseases, HF = heart failure, MR = Mendelian randomization, MI = myocardial infarction, OR = odds ratio, SNP = single nucleotide polymorphism.

Figure 5 presents the MR-derived causal effects of MI, CHD, HF and HTN. Figure 6 illustrates the SNP-exposure-outcome effect relationships. The IVW method suggested a marginal association between HF and hypothyroidism risk (OR = 1.006, 95% CI: 1.000–1.012, *P* = .040). A similar directional trend, albeit statistically nonsignificant, was observed in the (WM: OR = 1.007, 95% CI: 0.995–1.015, *P* = .072), simple mode (OR = 1.007, 95% CI: 0.995–1.020, *P* = .281), and weighted mode (OR = 1.008, 95% CI: 0.997–1.020, *P* = .202) analyses. However, none of the 5 MR methods supported a causal relationship between genetic susceptibility to MI (IVW: OR = 1.005, 95% CI: 0.995–1.015, *P* = .328), CHD (IVW: OR = 1.005, 95% CI: 0.998–1.011, *P* = .174), or HTN (IVW: OR = 1.004, 95% CI: 0.991–1.016, *P* = .546) and hypothyroidism risk.

### 3.4. Sensitivity analysis

**Figure 7.**
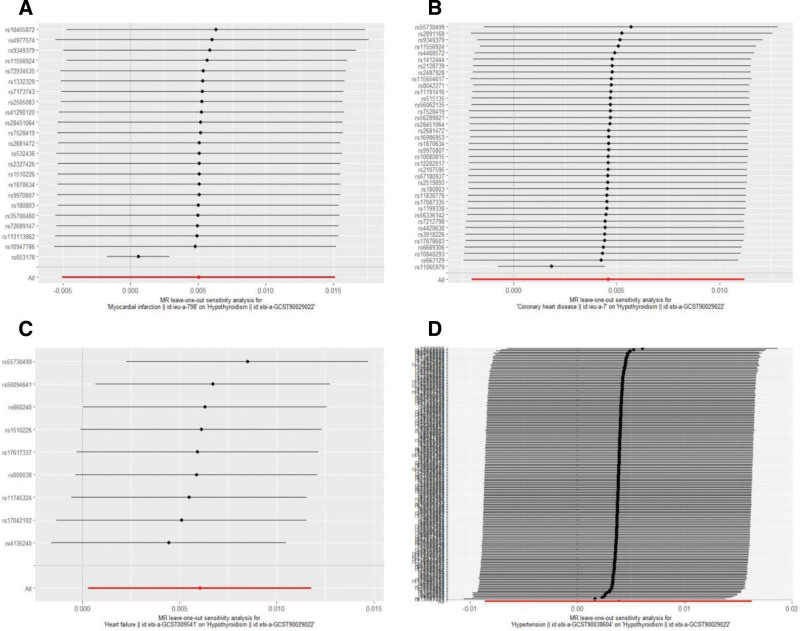
Leave-one-out sensitivity analysis between CVDs phenotype and hypothyroidism. (A) MI-hypothyroidism; (B) CHD-hypothyroidism; (C) HF-hypothyroidism; and (D) hypertension-hypothyroidism. Each line represents the causal estimate when sequentially excluding 1 SNP at a time. *X*-axis: estimated causal effect of CVDs phenotype on hypothyroidism risk. *Y*-axis: SNP identifier. The horizontal dashed line indicates the primary MR estimate. CHD = coronary heart disease, CVDs = cardiovascular diseases, HF = heart failure, MR = Mendelian randomization, MI = myocardial infarction, SNP = single nucleotide polymorphism.

Cochran’s *Q* test (Table 2) indicated heterogeneity among IVs for some associations (*P* < .05), prompting the use of IVW random-effects models. MR-Egger intercept tests (Table 2) revealed no evidence of horizontal pleiotropy (*P* > .05 for all), confirming the robustness of the IVs. Furthermore, leave-one-out sensitivity analysis (Fig. 7) did not identify outlier SNPs, further validating the stability of our results.

## 4. Discussion

This study employed a bidirectional TSMR design to investigate the potential causal relationships between hypothyroidism and CVDs from a genetic perspective, underscoring the importance of monitoring cardiovascular function in elderly individuals with hypothyroidism. The results demonstrated that hypothyroidism increases the risk of MI, CHD, HF, and HTN. However, in the reverse analysis, no causal associations were observed between the other 3 phenotypes of CVDs (MI, CHD, and HTN) and hypothyroidism, except for HF.

Regarding the link between hypothyroidism and CVDs, our findings confirm a causal relationship, consistent with prior research. Zhang^[30]^ in a large cohort study focusing on major cardiovascular and cerebrovascular adverse events post-percutaneous coronary intervention, reported that the hypothyroidism group exhibited significantly higher risks of major cardiovascular and cerebrovascular adverse events, HF, and MI compared to the euthyroid group. A cross-sectional Rotterdam study of 1149 women aged ≥55 years found that the hypothyroidism group had elevated risks of aortic atherosclerosis (OR = 1.9; 95% CI: 1.2–3.1) and MI (OR = 2.3; 95% CI: 1.3–4.2).^[31]^ Additionally, a large cross-sectional study of nearly 30,000 individuals revealed a linear relationship between systemic blood pressure and serum TSH levels.^[32]^ Animal studies further corroborate these findings. Wang^[33]^ observed impaired cardiac function, reduced ejection fraction, and elevated serum brain natriuretic peptide levels in hypothyroid rats. Tang^[34]^ demonstrated that prolonged hypothyroidism in adult mice led to coronary artery stenosis and severe progressive systolic dysfunction. Another study showed that hypothyroid dogs exhibited significantly larger infarct areas following induced MI compared to euthyroid controls.^[35]^ These studies collectively provide critical evidence for understanding the hypothyroidism-CVDs link.

The causal mechanisms underlying hypothyroidism and CVDs may involve. First is the lipid dysregulation, this study observed causal effect of hypothyroidism on CHD (OR = 2.78, *P* = .001) and MI (OR = 3.00, *P* = .002) aligns with the role of TH in lipid metabolism. Hypothyroidism reduces clearance of chylomicron remnants, suppresses cholesteryl ester transfer protein activity, and decreases hepatic and lipoprotein lipase activity, elevating total cholesterol, low-density lipoprotein cholesterol, and homocysteine levels.^[36,37]^ These lipid abnormalities accelerate atherosclerosis, inflammation, oxidative stress, and endothelial dysfunction, which are the key driver of CHD and MI, as reflected in the MR estimates. Besides, TSH also modulates endothelial function via TH receptor-α1 and TH receptor-β pathways,^[38]^ impairing vascular smooth muscle relaxation and nitric oxide availability, thereby increasing arterial stiffness and systemic vascular resistance.^[39]^ Meanwhile, the MR-derived causal association between hypothyroidism and HTN (OR = 1.10, *P* = .024) may be partly mediated by renin-angiotensin-aldosterone system dysfunction. Hypothyroidism suppresses renin synthesis, reduced renin activity decreases angiotensin II production, paradoxically causing sodium retention and plasma volume expansion via aldosterone-independent mechanisms, leading to diastolic HTN.^[40]^ Additionally, hypothyroidism reduces sarcoplasmic reticulum Ca²^+^-ATPase (SERCA2) expression, slowing Ca²^+^ reuptake into the sarcoplasmic reticulum and impairing myocardial relaxation.^[41]^ Phospholamban-mediated inhibition of SERCA2 prolongs cytoplasmic Ca²^+^ decay, increasing mitochondrial Ca²^+^ and oxidative stress.^[42]^ These mechanistically explains the the MR findings for HF (OR = 1.89, *P* = .009), as SERCA2 dysfunction and mitochondrial Ca²^+^ Overload is a hallmark of HF with preserved ejection fraction.

In reverse causality analysis, HF was marginal associated with an increased risk of hypothyroidism (OR = 1.006, 95% CI: 1.000–1.012, *P* = .040). The results indicated that HF patients had only a 0.6% increased risk of hypothyroidism. Even with statistically significant associations (*F*-statistic > 10, effectively avoiding bias from weak IVs) and consistent, robust sensitivity analyses, the absolute risk increment remained negligible – far below the clinical intervention threshold. Routine thyroid function screening or prophylactic treatment for HF patients is not recommended. However, this study provides genetic evidence supporting the “cardio-thyroid axis” hypothesis. Cross-sectional studies indicate that 30% of HF patients exhibit thyroid dysfunction, characterized by reduced myocardial triiodothyronine (T3) levels (low T3 syndrome), likely due to hypothalamic-pituitary-thyroid axis dysregulation, cytokine-mediated oxidative stress, and inflammation.^[43]^ Persistent low T3 levels exacerbate myocardial injury and remodeling, correlating with worse New York Heart Association functional class and higher mortality.^[44,45]^ Animal studies reveal reduced myocardial TSH receptor expression in CVD models, impairing signaling and inducing hypothyroidism.^[46,47]^ Altered TSH metabolism has also been reported in MI and HTN,^[47–49]^ though our study did not detect such associations. Further large-scale studies are needed to explore potential subgroup-specific causality.

## 5. Conclusion

The main innovation of this study is the use of a bidirectional TSMR design to analyze the causal relationship between multiple phenotypes of CVDs and hypothyroidism and to reduce the confounding factors and biases associated with bidirectional causality in observational studies. Also, the various analytical methods used in the study improved the precision and reproducibility of the findings. However, there are some limitations of our study. First, the scarcity of publicly aggregated GWAS data and the stringent screening conditions resulted in a relatively small sample size for analysis and some heterogeneity, but a random-effects model was used in this study to minimize the bias and error it introduces. Second, while our findings provide valuable insights, it is important to acknowledge that the genetic data used in this study were predominantly derived from individuals of European ancestry. This limitation may affect the generalizability of our results to other populations, as genetic effects, allele frequencies, and LD patterns can vary across ancestries. Future studies incorporating more diverse cohorts are needed to evaluate the transferability of these associations and ensure broader applicability. In addition, the specific mechanism of action between hypothyroidism and CVDs needs to be verified by further experimental studies. It is worth mentioning that artificial intelligence (AI) and machine learning can optimize CVDs risk prediction and personalized treatment by analyzing large-scale genetic and clinical data.^[50]^ In CVDs management, AI-assisted diagnostic tools have demonstrated high accuracy and efficiency. Future research could integrate AI technology to further explore underlying mechanisms and validate the generalizability of findings across diverse populations.

## Author contributions

**Conceptualization:** Jianing Chi, Yanhua Xue.

**Data curation:** Sheng Qiu.

**Methodology:** Wenqiang Ma.

**Project administration:** Yanhua Xue.

**Software:** Guanyu Yan.
